# Atraumatic Spontaneous Splenic Rupture Complicating Decompensated Chronic Liver Disease With Portal Hypertension: A Case Report

**DOI:** 10.1155/crra/3772906

**Published:** 2026-10-05

**Authors:** Prasanna Ghimire, Shreya Subedi, Ankit Mishra, Dipendra Khadka, Pragya Gautam Ghimire

**Affiliations:** ^1^ Department of Radiodiagnosis and Imaging Nepalgunj Medical College and Teaching Hospital Kohalpur Nepal; ^2^ Department of Internal Medicine Hepatology Unit Nepalgunj Medical College and Teaching Hospital Kohalpur Nepal; ^3^ Department of Pathology Nepalgunj Medical College and Teaching Hospital Kohalpur Nepal

**Keywords:** case report, cirrhosis, hemoperitoneum, portal hypertension, splenic rupture, splenomegaly

## Abstract

Atraumatic spontaneous splenic rupture (ASR) is a rare, potentially fatal condition accounting for fewer than 0.5% of all splenic ruptures, with reported mortality of up to 20% when diagnosis is delayed. We report a 63‐year‐old male with decompensated chronic liver disease and portal hypertension who presented with acute epigastric pain, progressive abdominal distension, and absolute constipation without antecedent trauma. Abdominal ultrasonography identified splenomegaly with a 5 × 6 cm anechoic intraparenchymal collection with subcapsular and perisplenic extension and moderate hemoperitoneum. Noncontrast computed tomography confirmed parenchymal laceration classified as AAST Grade III/WSES Class II. Contrast‐enhanced CT was deferred because of concurrent renal impairment. The patient remained hemodynamically stable and was successfully managed with conservative nonoperative management. This case highlights the importance of suspecting ASR in patients with hepatic disease and congestive splenomegaly without trauma, the utility of prognostic scoring in guiding management, and the role of contrast‐enhanced ultrasound as a nephroprotective imaging alternative when iodinated contrast is contraindicated.

## 1. Introduction

Atraumatic spontaneous splenic rupture (ASR) is defined as splenic parenchymal disruption without preceding mechanical trauma, accounting for fewer than 0.5% of all splenic ruptures and carrying a reported mortality of up to 20% when recognition or intervention is delayed [1]. The systematic review by Renzulli et al. of 845 cases classified 93% as pathological arising in a diseased spleen and 7% as idiopathic, across six etiological categories: neoplastic (30.3%), infectious (27.3%), noninfectious inflammatory (20.0%), drug‐related or treatment‐related (9.2%), mechanical (6.8%), and histologically normal spleen (6.4%) [1].

Congestive splenomegaly secondary to portal hypertension in chronic liver disease (CLD) is an important but underappreciated cause of pathological ASR [2, 3]. Because the presentation is frequently atypical, diagnosis requires a high index of suspicion [4]. We report a case of ASR complicating decompensated CLD and portal hypertension managed successfully with conservative nonoperative management (NOM), with a focused discussion of pathophysiology, imaging strategy, injury grading, and management principles. This case report was prepared in adherence to the CARE (CAse REport) guidelines.

## 2. Case Report

A 63‐year‐old male with a long‐standing history of heavy alcohol use corroborated by family members presented with a 2‐day history of acute epigastric pain, progressive abdominal distension, and absolute constipation (inability to pass either stool or flatus), with significant straining during defecation. He denied any recent trauma, falls, or abdominal injury. He was a known case of decompensated chronic liver disease (DCLD) complicated by portal hypertension. There was no significant family history of hematologic or hepatic disease. Three months prior, esophagogastroduodenoscopy had revealed large esophageal and gastric varices; endoscopic variceal ligation was performed and 2 units of packed red blood cells transfused for anemia. The patient was on diuretics with no recent large‐volume paracentesis and was not receiving any anticoagulant or antiplatelet therapy.

The patient appeared chronically ill and cachectic with jaundice and conjunctival pallor. The abdomen was mildly distended and dull to percussion throughout, without rebound tenderness, guarding, or rigidity. No caput medusae or palpable splenic tenderness was noted. Kehr′s sign (referred left shoulder pain due to diaphragmatic irritation) was absent. Vital signs were blood pressure 110/70 mmHg, heart rate 80 beats per min, temperature 37.0°C, oxygen saturation 96% on room air, and respiratory rate 22 breaths per min. The patient was hemodynamically stable throughout.

Complete blood count revealed hemoglobin 8.4 g/dL, hematocrit 29.3%, platelet count 1.96 × 10^5^/*μ*L, and white blood cell count 5170/*μ*L. The platelet count was within normal limits despite portal hypertension and splenomegaly, likely reflecting the acute nature of the presentation before the development of marked hypersplenism and thrombocytopenia. The patient had a known baseline serum creatinine of 1.60 mg/dL (mild chronic kidney disease). On admission, creatinine was 2.60 mg/dL, consistent with acute‐on‐chronic renal impairment likely related to prerenal azotemia and dehydration. With controlled intravenous hydration and avoidance of nephrotoxins, creatinine improved to 1.80 mg/dL by Day 4 and was 1.60 mg/dL at discharge.

The Child–Pugh score was 7 points (Class B), based on bilirubin, albumin, INR, the presence of mild ascites, and the absence of hepatic encephalopathy. The MELD score, calculated from creatinine, bilirubin, and INR, was 16. Both scores indicated moderate hepatic dysfunction with increased perioperative morbidity risk and supported a nonoperative approach provided hemodynamic stability was maintained. The underlying decompensated CLD carries a guarded long‐term prognosis; the immediate prognosis for the splenic rupture episode was favorable given hemodynamic stability and absence of active arterial extravasation.

Key laboratory parameters and prognostic scores are summarized in Table 1.

**Table 1:** Selected laboratory investigations and prognostic scores.

Parameter	Value	Parameter	Value
Hemoglobin	8.4 g/dL	Child–Pugh score	7 (Class B)
Hematocrit	29.3%	MELD score	16
Platelet count	1.96 × 10^5^/*μ*L	Serum creatinine	2.60 mg/dL
WBC count	5170/*μ*L	Serum urea	78.0 mg/dL
Total bilirubin	1.16 mg/dL	Serum albumin	2.9 g/dL
AST/ALT	36.7/12.36 U/L	INR	0.98
ALP	222.3 U/L	Serum sodium/potassium	138/3.6 mEq/L

Differential diagnoses considered included perforated hollow viscus, acute pancreatitis, spontaneous bacterial peritonitis, bowel obstruction, and splenic infarction. However, imaging findings of splenic parenchymal laceration with perisplenic hematoma and hemoperitoneum, together with the absence of trauma, established the diagnosis of ASR.

Abdominal ultrasonography demonstrated a shrunken liver with coarse parenchymal echotexture consistent with cirrhosis, a dilated portal vein at 13.3 mm, and periumbilical collateral vessels confirming portal hypertension. An ill‐defined anechoic collection measuring 5 × 6 cm was identified within the splenic parenchyma with subcapsular and perisplenic extension, with moderate free intraperitoneal fluid in the hepatorenal and splenorenal recesses and the pelvis, consistent with hemoperitoneum (Figure 1).

**Figure 1:**
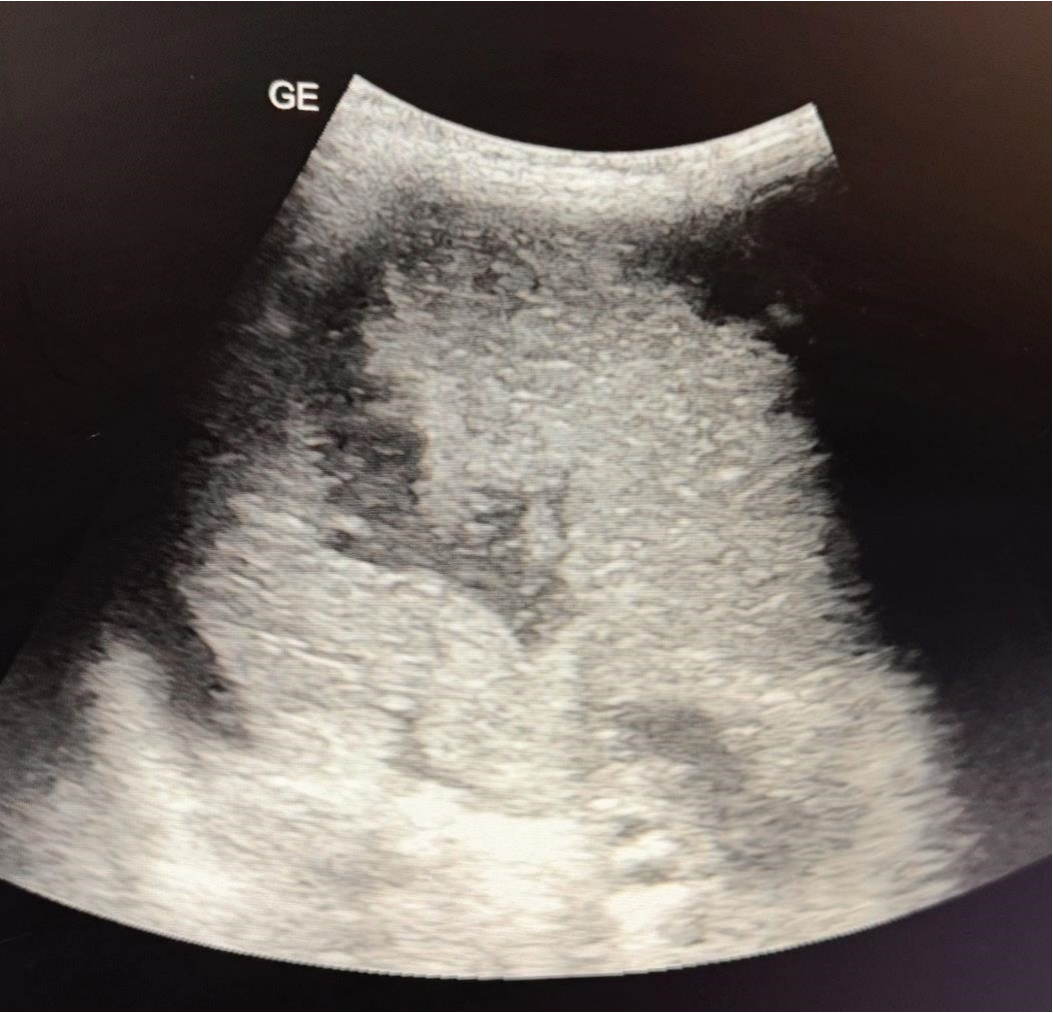
Abdominal ultrasonography in the longitudinal plane demonstrates an ill‐defined anechoic collection measuring 5 × 6 cm within the splenic parenchyma, extending into the subcapsular and perisplenic space, with moderate free intraperitoneal fluid consistent with hemoperitoneum.

Noncontrast computed tomography (NCCT) of the abdomen and pelvis confirmed multiple irregular linear hypodense areas extending from the capsular surface toward the splenic hilum, consistent with parenchymal lacerations, with an intraparenchymal hematoma with capsular disruption leading to subcapsular and perisplenic extension and mild‐to‐moderate hemoperitoneum. No injury to adjacent organs was identified (Figures 2A,B). Imaging did not demonstrate features of mechanical bowel obstruction or fecal impaction.

**Figure 2:**
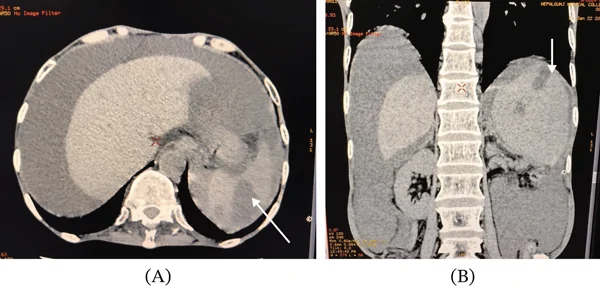
(A) Noncontrast axial CT image demonstrating multiple irregular linear hypodense areas extending from the capsular surface toward the splenic hilum, consistent with parenchymal lacerations (arrow), with associated mild‐to‐moderate hemoperitoneum. (B) Noncontrast coronal CT image demonstrating splenic laceration (arrow) and tortuous collateral vessels at the splenic hilum, consistent with portal hypertension.

Contrast‐enhanced CT (CECT) was deferred given acute‐on‐chronic renal impairment (creatinine 2.60 mg/dL) and clinical stability. Renal impairment is a relative, not absolute, contraindication to CECT. In this hemodynamically stable patient with stable serial hemoglobin and improving clinical status, the immediate diagnostic yield of CECT was balanced against the risk of worsening renal function. This decision represents an acknowledged diagnostic limitation: vascular injuries such as pseudoaneurysms or active arterial extravasation, which would upgrade the AAST grade and potentially alter management, cannot be excluded on NCCT alone. Sustained hemodynamic stability, the absence of a falling hemoglobin trend, and no clinical deterioration collectively provided sufficient reassurance to proceed with conservative management under close surveillance. Had the patient become hemodynamically unstable, developed a falling hemoglobin trend, or shown signs of peritonitis, emergency CECT (with appropriate volume expansion and renal‐protective measures) or direct catheter angiography/angioembolization would have been performed without delay.

When CECT is contraindicated and vascular injury assessment is required, contrast‐enhanced ultrasound (CEUS) is a validated nephroprotective alternative with reported sensitivity of up to 95% for parenchymal laceration and real‐time detection of arterial extravasation, employing microbubble agents excreted via pulmonary respiration with no nephrotoxic risk. CEUS was not performed here for two reasons: NCCT had already established the diagnosis in a clinically stable patient, and CEUS was in any case not available at our institution at the time of presentation. It should be considered when renal impairment and the need for vascular characterization coexist and the modality is accessible. The injury was graded AAST Grade III (intraparenchymal hematoma exceeding 5 cm with capsular laceration) and WSES Class II (stable patient, Grade III injury), for which ICU monitoring with a low threshold for escalation is recommended.

General surgery and hepatology were consulted jointly. Given hemodynamic stability, the absence of peritoneal signs, and the high operative risk reflected by Child–Pugh Class B and MELD 16, conservative NOM was elected. The patient was admitted to the ICU for continuous monitoring, intravenous fluid support, analgesics, serial abdominal examination, strict bed rest, and hemoglobin measurement every 4 to 8 h. Serial hemoglobin remained stable without transfusion requirement (8.4 g/dL on admission, 8.2 g/dL at 24 h, 8.7 g/dL at 48 h, 8.8 g/dL at 72 h, and 9.2 g/dL at discharge), with no hemodynamic deterioration or worsening abdominal findings, allowing transfer to the general ward after 72 h. No escalation of intervention was required; the initial conservative strategy was maintained throughout the admission without modification. Adherence to bed rest and monitoring was assessed through nursing observation; the patient tolerated conservative management without adverse events or complications. Prior to discharge, a repeat transabdominal ultrasound was performed, which was not remarkable and demonstrated stability of the perisplenic collection without new fluid accumulation, arguing against a delayed or “double‐rupture” event. At short‐term clinical follow‐up, the patient remained hemodynamically stable with improvement in abdominal pain and no evidence of recurrent bleeding (Table 2). Because the patient was managed nonoperatively, no splenic tissue was available for histopathological examination.

**Table 2:** Timeline of events.

Time	Clinical event
3 months before presentation	Esophagogastroduodenoscopy revealed large esophageal and gastric varices; endoscopic variceal ligation performed with transfusion of two units packed red blood cells
2 days before admission	Acute epigastric pain, progressive abdominal distension, and absolute constipation developed
Day of admission	Ultrasonography and NCCT established the diagnosis of atraumatic spontaneous splenic rupture
ICU stay (72 h)	Conservative nonoperative management with serial monitoring
Discharge	Hemodynamically stable without transfusion requirement; outpatient follow‐up advised

## 3. Discussion

ASR was first described by Rokitansky in 1861 in a patient with leukemic infiltration. True idiopathic ASR requires the Orloff–Peskin criteria: no antecedent trauma, no systemic splenic disease, no perisplenic adhesions, histologically normal splenic parenchyma, and normal coagulation extended by Crate and Payne in 1991 to include the absence of viral seroconversion [5, 6]. The present case does not meet these criteria: documented DCLD with portal hypertension and congestive splenomegaly violates the requirements for absence of systemic disease and histological normality. Normal WBC, INR, and the absence of lymphadenopathy or systemic B symptoms exclude infectious and neoplastic etiologies, supporting congestive splenomegaly as the operative mechanism [1, 6, 7].

In cirrhosis, sinusoidal fibrosis increases intrahepatic resistance and raises splenic venous pressure, producing chronic passive congestion [2, 3]. This leads to sinusoidal dilatation, reticular cell hyperplasia, and progressive capsular and perivascular fibrosis, as documented histologically by Li et al. [8]. The resulting loss of splenic compliance stretches the capsule to its limit. A transient rise in intra‐abdominal pressure such as straining during constipation, as in this case can then trigger capsular micro‐tear, hematoma formation, and free peritoneal hemorrhage [2, 3, 7, 9]. In this patient, absolute constipation for 2 days led to significant straining during defecation. Straining increases intra‐abdominal and intrasplenic venous pressure, which in a chronically congested, noncompliant spleen likely acted as the immediate mechanical trigger for capsular micro‐tear and subsequent rupture. Additionally, endoscopic variceal ligation performed 3 months prior may have altered gastroesophageal collateral flow, potentially shifting venous pressure dynamics into the short gastric and splenic venous systems.

The classic triad of acute left upper quadrant pain, hemodynamic instability, and Kehr′s sign was absent here. The patient presented with epigastric pain, was hemodynamically stable, and had no shoulder tip radiation [1, 4]. Atypical presentations occur when hemorrhage is slow, the hematoma is capsule‐contained, or peritoneal irritation is attenuated by ascitic fluid, which is all plausible in portal hypertension [1, 4, 10, 11]. This case reaffirms that the absence of the classic triad should not exclude ASR when hepatic disease and splenomegaly are present.

Ultrasonography is the most accessible first‐line modality and was diagnostic in this case. CECT is the gold standard for injury grading, detecting active arterial extravasation and vascular lesions that inform management escalation [5, 10]. FAST allows rapid bedside detection of hemoperitoneum in unstable patients. Deferring CECT was clinically justified in this case by renal impairment and hemodynamic stability, but carries an acknowledged limitation: vascular injuries that would upgrade the AAST grade and potentially mandate splenic artery embolization (SAE) cannot be excluded on NCCT alone [10]. The decision was supported by the absence of hemodynamic deterioration, a stable hemoglobin trend, and no peritoneal signs, with an explicit plan to obtain vascular imaging if any parameter changed. This reflects the principle that in hemodynamically stable patients without clinical deterioration, the risk of contrast‐induced nephropathy in the setting of significant renal impairment can outweigh the benefit of complete vascular characterization [6, 11]. When CECT is contraindicated and vascular assessment is clinically required, CEUS offers a nephroprotective alternative with sensitivity up to 95% for parenchymal injury and real‐time extravasation detection using microbubble agents with no nephrotoxic risk [5, 6]. In patients with cirrhosis and portal hypertension, rupture of a splenic artery aneurysm or pseudoaneurysm is an important differential diagnosis for hemoperitoneum. The hyperdynamic splanchnic circulation predisposes to medial degeneration and aneurysm formation, and rupture may present with clinical features overlapping parenchymal splenic rupture [12]. In our patient, careful review of the noncontrast CT did not show coarse vascular calcification, focal arterial dilation, or a high‐attenuation aneurysmal sac along the main splenic artery or its hilar branches. However, noncontrast CT cannot definitively exclude small pseudoaneurysms or active arterial extravasation; this represents a key diagnostic limitation. Had the patient become hemodynamically unstable or shown a falling hemoglobin trend, urgent vascular imaging (CECT or catheter angiography) would have been pursued.

NOM is appropriate in hemodynamically stable patients without peritonitis or active arterial extravasation, and requires ICU admission, bed rest, and serial hemoglobin monitoring [10, 11, 13, 14]. It is not universally applicable: age ≥ 40 years, high‐grade injury, and large hemoperitoneum increase the risk of failure and mandate close surveillance [10, 14, 15]. In this case, the Child–Pugh Class B/MELD 16 profile made the alternative emergency splenectomy particularly hazardous given the associated risks of hemorrhage from hilar collateral varices, acute portal vein thrombosis, and acute‐on‐chronic liver failure [1, 9].

When NOM is insufficient, SAE is an effective minimally invasive option with a reported failure rate of 5.3% and postembolization mortality of 0.08% in a systematic review of 3835 patients [13]. Splenectomy remains the definitive intervention for refractory hemodynamic instability and must be accompanied by vaccination against *Streptococcus pneumoniae, Neisseria meningitidis*, and *Haemophilus influenzae* type b to prevent overwhelming postsplenectomy infection. [1, 5] A major strength of this report is the detailed imaging‐based diagnostic and management approach in a patient with concurrent renal impairment limiting contrast‐enhanced evaluation. Limitations include the absence of vascular characterization with CECT or CEUS and limited long‐term follow‐up. A comparative summary of the present case alongside previously published cases of atraumatic splenic rupture in cirrhosis/portal hypertension and other reported etiologies is presented in Table 3.

**Table 3:** Comparative summary of published cases of atraumatic splenic rupture in cirrhosis/portal hypertension and other reported etiologies.

Author and year	Age/sex	Primary etiology/underlying condition	AAST grade/WSES class	Management strategy	Outcome
Li et al. (2020) [6]	56/M	Cirrhosis, portal hypertension, splenomegaly, hypersplenism, and intracystic hemorrhage	Not graded in original report	Emergency splenectomy	Recovered
Perez et al. (2023) [4]	57/F	Hypertension; no underlying cause identified	Not graded in original report	Splenic artery embolization followed by splenectomy	Recovered
Santarelli et al. (2024) [11]	73/F	Idiopathic atraumatic splenic rupture; no underlying splenic or systemic pathology identified	Not graded in original report	Emergency splenectomy	Recovered
Mustafiz et al. (2025) [9]	Male, late 30s	Cirrhosis, portal hypertension, and Q fever	Not graded in original report	Emergency splenectomy	Recovered
Present case (2026)	63/M	Alcohol‐related decompensated chronic liver disease, portal hypertension, and congestive splenomegaly	AAST Grade III/WSES Class II	Conservative nonoperative management	Recovered

## 4. Conclusion

This case demonstrates that ASR can complicate portal hypertension without trauma and without the classic diagnostic triad. The combination of ultrasonography, NCCT, and prognostic scoring was sufficient to establish diagnosis, grade injury, and guide management in a patient in whom CECT was relatively contraindicated by renal impairment. Conservative NOM, when applied to appropriately selected hemodynamically stable patients with close surveillance and an explicit escalation plan, offers a safe strategy that avoids the compounded risks of emergency surgery in decompensated liver disease.

**Nomenclature**
AAST: American Association for the Surgery of Trauma
CECT: contrast‐enhanced computed tomography
CEUS: contrast‐enhanced ultrasound
CLD: chronic liver disease
CT: computed tomography
DCLD: decompensated chronic liver disease
EVL: endoscopic variceal ligation
ICU: intensive care unit
INR: international normalized ratio
MELD: model for end‐stage liver disease
NCCT: noncontrast computed tomography
NOM: nonoperative management
SAA: splenic artery aneurysm
USG: ultrasonography
WSES: World Society of Emergency Surgery

## Author Contributions

P.G.: conceptualization, investigation, writing—original draft, supervision. S.S.: investigation, writing—original draft. A.M.: data curation, writing—review and editing. D.K.: clinical management, writing—review and editing. P.G.G.: pathology review, writing—review and editing.

## Funding

No funding was received for this manuscript.

## Consent

Written informed consent was obtained from the patient for publication of this case report and any accompanying images. No identifiable patient information is included in the images or clinical data presented.

## Conflicts of Interest

The authors declare no conflicts of interest.

## Patient Perspective

The patient expressed relief that surgical intervention could be avoided and reported satisfaction with the conservative management and clinical improvement during hospitalization.

## Supporting information


**Supporting Information** Additional supporting information can be found online in the Supporting Information section. Data S1: CARE checklist of information to include when writing a case report.

## Data Availability

The data that support the findings of this study are available on request from the corresponding author. The data are not publicly available due to privacy or ethical restrictions.
