# Supplementary material for: Atraumatic Spontaneous Splenic Rupture Complicating Decompensated Chronic Liver Disease With Portal Hypertension: A Case Report
Source: Case Rep Radiol. 2026 Oct 5;2026:3772906. doi: 10.1155/crra/3772906 (PMC13636672; doi:10.1155/crra/3772906)
Supplement: Supplementary file 1 [file CRRA-2026-3772906-s001.pdf]

CARE
case report guidelines

CARE Checklist of information to include when writing a case report

(cc) BY-NC-ND

☑

Topic	Item	Checklist item description	Reported on Line
Title	1	The diagnosis or intervention of primary focus followed by the words “case report”	1-2
Key Words	2	2 to 5 key words that identify diagnoses or interventions in this case report, including "case report"	52-53
Abstract (no references)	3a	Introduction: What is unique about this case and what does it add to the scientific literature?	32-35
	3b	Main symptoms and/or important clinical findings	35-42
	3c	The main diagnoses, therapeutic interventions, and outcomes	39-48
	3d	Conclusion—What is the main “take-away” lesson(s) from this case?	47-51
Introduction	4	One or two paragraphs summarizing why this case is unique (**may include references**)	55-68
Patient Information	5a	De-identified patient specific information.	69-78
	5b	Primary concerns and symptoms of the patient.	69-77
	5c	Medical, family, and psycho-social history including relevant genetic information	69-83
	5d	Relevant past interventions with outcomes	79-83, 187-207
Clinical Findings	6	Describe significant physical examination (PE) and important clinical findings.	84-91
Timeline	7	Historical and current information from this episode of care organized as a timeline	table 2
Diagnostic Assessment	8a	Diagnostic testing (such as PE, laboratory testing, imaging, surveys).	92-117
	8b	Diagnostic challenges (such as access to testing, financial, or cultural)	141-168
	8c	Diagnosis (including other diagnoses considered)	118-140
	8d	Prognosis (such as staging in oncology) where applicable	104-110
Therapeutic Intervention	9a	Types of therapeutic intervention (such as pharmacologic, surgical, preventive, self-care)	173-186
	9b	Administration of therapeutic intervention (such as dosage, strength, duration)	141-186
	9c	Changes in therapeutic intervention (with rationale)	187-201
Follow-up and Outcomes	10a	Clinician and patient-assessed outcomes (if available)	193-201
	10b	Important follow-up diagnostic and other test results	193-201
	10c	Intervention adherence and tolerability (How was this assessed?)	190-193
	10d	Adverse and unanticipated events	190-193
Discussion	11a	A scientific discussion of the strengths AND limitations associated with this case report	274-277
	11b	Discussion of the relevant medical literature **with references**.	208-273
	11c	The scientific rationale for any conclusions (including assessment of possible causes)	208-273
	11d	The primary “take-away” lessons of this case report (without references) in a one paragraph conclusion	308-310
Patient Perspective	12	The patient should share their perspective in one to two paragraphs on the treatment(s) they received	293-296
Informed Consent	13	Did the patient give informed consent? Please provide if requested	Yes ☑ No ☐
